# Functional Magnetic Resonance Imaging Signal Typically Viewed as “Noise” Has Clinical Relevance in Psychiatry

**DOI:** 10.1016/j.bpsgos.2026.100720

**Published:** 2026-03-06

**Authors:** Julia C. Welsh, Cole Korponay, Tianye Zhai, Justine A. Hill, Betty Jo Salmeron, Blaise B. Frederick, Amy C. Janes

**Affiliations:** a Neuroimaging Research Branch, National Institute on Drug Abuse, Intramural Research Program, National Institutes of Health, Baltimore, Maryland; b Brain Imaging Center, McLean Hospital, Belmont, Massachusetts; c Department of Psychiatry, Harvard Medical School, Boston, Massachusetts

**Keywords:** Cue reactivity, fMRI, Low-frequency oscillations, Methylphenidate, Nicotine, Physiology

## Abstract

**Background:**

Functional magnetic resonance imaging (fMRI) aims to identify biomarkers of neuropsychiatric illnesses, including substance use disorders; however, fMRI’s clinical utility remains limited. One underexplored aspect of the fMRI signal is the systemic low-frequency oscillation (sLFO), a global physiological signal historically treated as noise but linked to vascular and autonomic function.

**Methods:**

Across 4 independent datasets, we used Regressor Interpolation at Progressive Time Delays analyses to extract the sLFO from resting-state and task-based fMRI data. We evaluated relationships between sLFO amplitude and acute and chronic indices of nicotine use. We further assessed the effects of nicotine and methylphenidate on the sLFO and related task performance.

**Results:**

In individuals who smoked tobacco, higher average sLFO amplitude during cigarette cue exposure was negatively associated with nicotine dependence (*n* = 64; *r* = −0.32, *p* = .009), whereas a rise in the sLFO across the task was negatively associated with greater cue-induced craving (*r* = −0.31, *p* = .014). sLFO amplitude was reduced during chronic nicotine use versus abstinence (*n* = 65; *p* < .001). Relative to placebo, acute single-dose methylphenidate administration also reduced the sLFO in healthy control participants (*n* = 58; *p* = .001). Both acute methylphenidate- and nicotine-induced reductions in sLFO amplitude were associated with improved cognitive task performance.

**Conclusions:**

These findings demonstrate that the sLFO encodes biologically meaningful information related to substance use, consistent with its role as an index of physiological arousal. Importantly, because the sLFO can be extracted directly from existing fMRI datasets, it offers a powerful and complementary approach to enhance the clinical relevance of fMRI research beyond substance use.

## Plain Language Summary

In this study, we investigated whether an often-overlooked signal in brain scans can help us understand substance use disorders better. Unlike typical brain imaging signals that reflect brain activity, this signal reflects physiology of the whole body. Because substance use affects both the brain and body, we tested whether this signal relates to nicotine use. We found that the whole-body signal was linked to drug dependence, craving, and withdrawal and that the signal changes with drug administration in ways that relate to cognitive performance. These results suggest that this signal carries useful clinical information for studying substance use and mental health.

Functional magnetic resonance imaging (fMRI) is central to efforts guiding personalized, neurobiologically informed treatments for neuropsychiatric illnesses, including substance use disorders. However, fMRI’s ability to explain individual differences in mental health remains limited. While fMRI predominantly measures neural activity, drugs of abuse also produce robust physiological effects. For example, nicotine is a well-documented vasoconstrictor and psychostimulant, affecting vascular function and physiological arousal (1). This suggests that physiological signals in fMRI—often treated as confounds—may instead be leveraged to explain clinically relevant differences.

The blood oxygen level–dependent (BOLD) signal in fMRI reflects both neural and physiological processes. While neuronal contributions are usually studied, a low-frequency component—the systemic low-frequency oscillation (sLFO, ∼0.009–0.15 Hz)—carries direct physiological relevance. The sLFO is a pervasive global signal driven largely by physiological processes such as blood flow, respiration, and vasomotion (2,3) and inversely tracks physiological arousal; lower sLFO amplitude corresponds to higher arousal as indexed by heart rate variability, respiration, and pupil diameter (4,5). This is true during wakeful fluctuations and across transitions toward sleep, indicating that reductions in arousal alter sLFO dynamics (4). Parallel recordings further demonstrate close correspondence between sLFO patterns in the brain and fingertip (6,7), underscoring its systemic physiological basis.

Unlike low-frequency oscillations linked to neuronal activity through neurovascular coupling (8,9), the sLFO is a single global signal that propagates along the vasculature with region-specific time delays (2,7,10). Despite this physiological relevance (3), the sLFO is typically viewed as noise (4), which can be discarded without disrupting well-characterized brain signals (4,6,10,11). The dismissal of the sLFO as noise mirrors long-standing debates about the global signal, which is similarly treated as noise despite evidence that it contains meaningful information (12, 13, 14, 15). For example, a growing body of research demonstrates that fluctuations in the sLFO, global signal, and related physiological signals such as heart rate, respiration, and vasomotion track changes in arousal, sleep state, anxiety, and diurnal rhythms (4,5,16, 17, 18, 19, 20, 21). While the global signal has been linked to arousal, its interpretation is complicated by the fact that it comprises not only the sLFO (summed over a range of delay times) but also a mixture of noise sources, including head motion and scanner instabilities. Unlike the global signal, the sLFO is a cleaner, time-lagged physiological signal that can be extracted from standard fMRI, providing both improved neural estimates and a direct physiological marker.

Given that arousal, autonomic function, and vascular physiology are disrupted in substance use and are modulated by drug cues and withdrawal, the sLFO may provide a sensitive physiological marker for craving, behavior, and drug effects. Here, we tested this across 4 cohorts, examining how the sLFO responds to 1) smoking cues in individuals who smoke cigarettes, 2) nicotine abstinence in individuals who smoke cigarettes, and 3) acute psychostimulant administration in healthy control individuals. We further assessed how the sLFO relates to nicotine dependence, craving, and task performance. These analyses evaluate whether the sLFO functions as a physiologically grounded marker that helps explain individual differences in addiction.

## Methods and Materials

We analyzed 4 independent cohorts (detailed in the Supplement). Participants were healthy and tested negative for pregnancy and acute drug/alcohol use (except nicotine, where noted). Studies were approved by local institutional review boards.

### Analysis Overview

**Figure 1:**
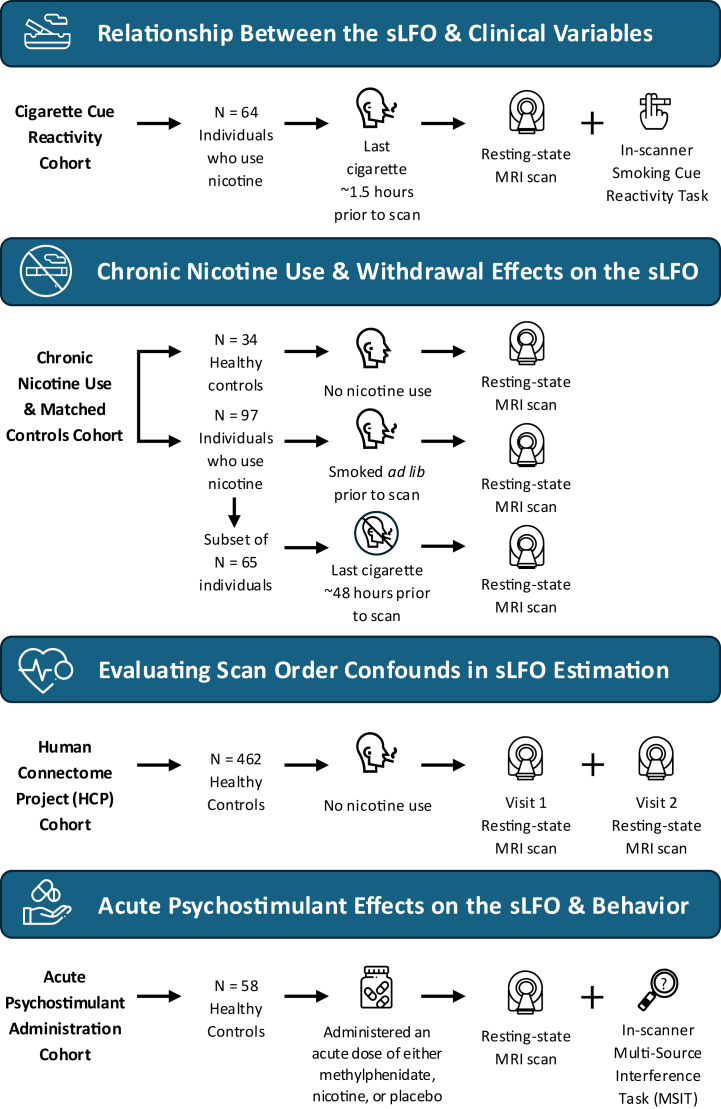
Overview of the 4 independent cohorts analyzed. Schematic illustration of the study design highlighting the 4 cohorts used to examine complementary aspects of systemic low-frequency oscillation (sLFO) physiology: 1) the Cigarette Cue Reactivity Cohort, assessing task-evoked physiological fluctuations and their relationship to nicotine-related clinical measures; 2) the Chronic Nicotine Use and Matched Controls Cohort, comparing resting-state sLFO features in individuals who did and did not smoke cigarettes and testing the impact of short-term nicotine abstinence in a subset of individuals who smoked cigarettes; 3) the HCP (Human Connectome Project) Cohort, evaluating potential confounds related to scan order; and 4) the Acute Psychostimulant Administration Cohort, testing drug-induced modulation of sLFO activity and associated behavioral effects. This diagram depicts the complementary roles of each cohort and how, together, they inform our overarching investigation of sLFO physiology. MRI, magnetic resonance imaging.

Different cohorts were used to test complementary aspects of sLFO physiology (Figure 1). First, in the Cigarette Cue Reactivity Cohort, we sought to determine whether exposure to smoking cues induces sLFO shifts and is associated with nicotine dependence and craving. Second, in the Chronic Nicotine Use and Matched Controls Cohort, we sought to determine how the sLFO at rest compares in individuals who do and do not smoke and whether the sLFO is impacted by nicotine abstinence in individuals who smoke. Third, in the HCP (Human Connectome Project) Cohort, we tested whether scan order influences sLFO estimates. Finally, in the Acute Psychostimulant Administration Cohort, we assessed whether psychostimulants modulate the sLFO in healthy control individuals and whether such changes relate to task performance. Cohort-specific analytic choices followed each parent study’s conventions and do not affect central conclusions.

### Participants

#### Cigarette Cue Reactivity Cohort

This cohort comprised 64 individuals who chronically smoked cigarettes (25 female; age, mean ± SD = 29.19 ± 6.93 years), with nicotine dependence defined by the Fagerström Test for Nicotine Dependence (FTND) scale (average = 4.77 ± 2.02) (22). Before scanning, average expired carbon monoxide (CO) was 20.39 ± 12.51 parts per million (ppm). Participants smoked one of their own cigarettes 1.5 hours before the scan.

#### Chronic Nicotine Use and Matched Controls Cohort

This cohort comprised 97 individuals who did (42 female; age, mean ± SD = 48.23 ± 10.84 years) and 34 individuals who did not (12 female; age, mean ± SD = 46.24 ± 9.45 years) chronically smoke cigarettes. Participants in the smoking group had an average FTND score of 4.59 ± 1.94 and an average expired CO of 23.94 ± 11.96 ppm at baseline. Participants who did not use nicotine underwent one scan. Participants who used nicotine underwent at least one scan while sated, and a subset of these individuals (*n* = 65; 28 female; age, mean ± SD = 47.85 ± 11.05 years) underwent a second scan following 48 hours of nicotine abstinence, confirmed by a reduced CO (2.63 ± 1.90 ppm; *t*_64_ = 14.79, *p* < .001).

#### HCP Cohort

This cohort comprised 462 participants from the Young Adult dataset who we previously assessed given they have no current or family history of psychiatric illness or substance use (281 female; age, mean ± SD = 28.66 ± 3.65 years) (4,23). Each participant was scanned on 2 separate days, with 2 scans per day recorded using different phase-encode directions, right to left (RL) and left to right (LR).

#### Acute Psychostimulant Administration Cohort

This cohort comprised 58 healthy control participants (41 female; age, mean ± SD = 31.17 ± 9.37 years). Participants had no lifetime history of substance use disorders and no substance abuse for at least 2 years before participating. Participants were scanned following administration of 7 mg nicotine, 20 mg methylphenidate, or placebo—each timed to drug peak—using a randomized, counterbalanced, double-blinded design.

### fMRI Acquisition

Resting fMRI data were collected in all cohorts, during a cue reactivity task in the Cigarette Cue Reactivity Cohort and during the Multi-Source Interference Task (MSIT) in the Acute Psychostimulant Administration Cohort. Acquisition and preprocessing details are provided in the Supplement.

### Cigarette Cue Reactivity Task

The cigarette cue reactivity task (24,25) consisted of five 5-minute runs presenting 10 smoking, 10 neutral, and 2 target images in pseudorandom order, with no more than 2 of the same type in a row. Images were presented for 4 seconds followed by a 6- to 14-second (mean = 10 seconds) jittered intertrial interval consisting of a black screen and a white fixation cross. Smoking images depicted tobacco use; neutral images were matched in content but without smoking cues. Target images (animals) required a button press to maintain attention. Subjective nicotine craving was measured pre- and postscan using the Questionnaire of Smoking Urges (QSU) Brief Form (26), with cue-induced craving calculated as postscan − prescan QSU scores.

### Multi-Source Interference Task

Participants were shown 3-digit stimuli and were asked to indicate via button-press the position of the number that differed from the other numbers presented (27). During congruent trials, the identity and position of the target numbers were consistent. During incongruent trials, the identity and position of the target numbers were inconsistent. The task consisted of 3 blocks, with each block consisting of 24 stimuli split evenly between trial types. Stimuli were presented for 2 seconds followed by a 4- to 8-second jittered intertrial interval in increments of 500 ms. Task accuracy and reaction time were recorded.

### Data Analysis

#### sLFO Identification

The sLFO was modeled and quantified using Regressor Interpolation at Progressive Time Delays (RIPTiDe) (https://github.com/bbfrederick/rapidtide). This method isolates low-frequency fluctuations (0.009–0.15 Hz) and estimates voxelwise blood arrival times via cross-correlation to create lag maps capturing the temporal propagation of the sLFO across the brain (2,4,10,28). These maps reflect vascular transit delays distinct from neural hemodynamic responses (28,29).

A refined sLFO reference regressor is then generated by iteratively bootstrapping from the sLFO-filtered global mean BOLD signal. This process uses cross-correlation to determine the arrival times of the sLFO, aligning voxel time courses and applying principal component analysis to extract the reference regressor. We created voxel-specific regressors by adjusting the refined sLFO regressor according to the blood arrival times for each voxel. For each subject and scan condition, a map was generated demonstrating the average sLFO amplitude for every voxel. The reported sLFO findings refer to the brainwide average sLFO amplitude across all voxels, whereas the voxelwise sLFO refers to the regional sLFO. In the Cigarette Cue Reactivity Cohort, fMRI data from the smoking > neutral contrast with and without the sLFO included were compared. A linear regression model was used to filter, fit, and eliminate the signal from the fMRI data, removing physiological noise without causing spurious negative correlations between regions (10).

### Cigarette Cue Reactivity Task Analyses

The cue reactivity analysis was conducted using the FMRIB software library (FSL) (http://www.fmrib.ox.ac.uk/fsl) using methods outlined in the Supplement and described previously (24,25). The smoking > neutral contrast was corrected for multiple comparisons using a cluster threshold of *z* = 3.1, *p* < .05. This analysis was independently conducted twice, before and after RIPTiDe removed the sLFO. The correlations between the smoking > neutral activation patterns for analyses with and without the sLFO were calculated using FSL’s FSLCC command.

Given prior work demonstrating enhanced smoking > neutral reactivity in the anterior insula and default mode network (DMN) (30), we conducted follow-up region-of-interest (ROI) analyses evaluating the insula and DMN from data with the sLFO removed (Figure S1) (31). ROI changes in smoking > neutral activation over the 5 task runs were calculated using a repeated-measures analysis of variance (ANOVA). A Pearson correlation was used to assess the relationship between the average ROI activation and average sLFO across the 5 runs of the cue reactivity task.

### Change in the sLFO Across Rest and Cue Reactivity Runs

A repeated-measures ANOVA comparing the brainwide average sLFO was conducted considering scan block (rest, cue blocks 1–5). Post hoc 2-sided paired *t* tests were conducted to define directionality.

### Relationship Between the sLFO, Nicotine Dependence, and Cue-Induced Craving

Separate Pearson correlations were examined between FTND scores and the sLFO amplitude measured during rest and during the 5 runs of the cue reactivity task. We also tested the association between cue-induced craving (pre- vs. postcue reactivity) and cue-induced change in the sLFO, calculated as the difference in the brainwide average sLFO between the last and first cue block. Because the FTND measures a static trait, and cue-induced craving reflects dynamic changes, we correlated FTND scores with the average sLFO during the cue reactivity task and cue-induced craving with cue-related sLFO changes. These correlations were treated as a priori, nonindependent tests based on prior work on cue-induced arousal and addiction-related physiological responses; therefore, no additional correction for multiple comparisons was applied.

### sLFO, Chronic Nicotine Use, Withdrawal, and Acute Psychostimulant Administration

In the Chronic Nicotine Use and Matched Controls Cohort, the brainwide average resting-state sLFO was compared across conditions using 2-tailed, 2-sample *t* tests. Comparisons included healthy control individuals versus individuals who smoked during nicotine satiety or abstinence. Among individuals who smoked cigarettes, the brainwide average sLFO at rest during nicotine abstinence versus satiety was compared using a paired-samples 2-tailed *t* test. In the Acute Psychostimulant Administration Cohort, the brainwide average sLFO was analyzed separately at rest and during the MSIT. The placebo versus acute nicotine/methylphenidate administration conditions were compared using 2-sided paired-sample *t* tests. To parse out regional differences in the voxelwise sLFO at rest between conditions, 2-sided paired-sample *t* tests were conducted using whole-brain voxelwise sLFO amplitude maps. Statistical significance was defined at the voxel level *p* < .001 and randomization-based multiple comparison correction at cluster level α < .05, NN1 (facewise nearest neighbor) (*3dttest++*, AFNI).

### Potential Confound of Scan Day Order

In the Chronic Nicotine Use and Matched Controls Cohort, the sated and abstinent conditions were not randomly counterbalanced across scan sessions. To test whether the sLFO changed as a function of scan day order, we conducted day 1 versus day 2 analyses of the brainwide average and voxelwise sLFO strength in healthy control participants in the HCP Cohort. Given that the HCP collected 2 scan sessions per day using different encoding directions (RL and LR), we quantified day-order effects using a one-sample *t* test comparing the average change in brainwide average sLFO between the first run of each day (i.e., RL1 and LR2) and the second run of each day (i.e., LR1 and RL2). Given high rates of family relatedness among the subjects in the HCP Young Adult dataset, all analyses were also corrected for covariance in family structure (i.e., the presence of siblings and twins in the study) using Permutation Analysis of Linear Models (32) with multilevel exchangeability blocks (33). For the voxelwise sLFO analysis, this difference was tested using a one-sided one-sample *t* test using FSL randomise with 5000 permutations, applying a threshold of *p* < .05 with threshold-free cluster enhancement.

### Multi-Source Interference Task Analyses

Drug effects on the sLFO and task performance were calculated by subtracting the placebo values from drug condition values for the brainwide average sLFO, reaction time, and accuracy. Drug-induced change in brainwide average sLFO was then correlated with drug-induced task changes. Because MSIT accuracy and reaction time reflect nonindependent, theory-driven measures of task performance, these correlations were treated as planned tests without further multiple-comparisons correction.

## Results

### sLFO Changes Across Resting-State and Cue Reactivity Scans

**Figure 2:**
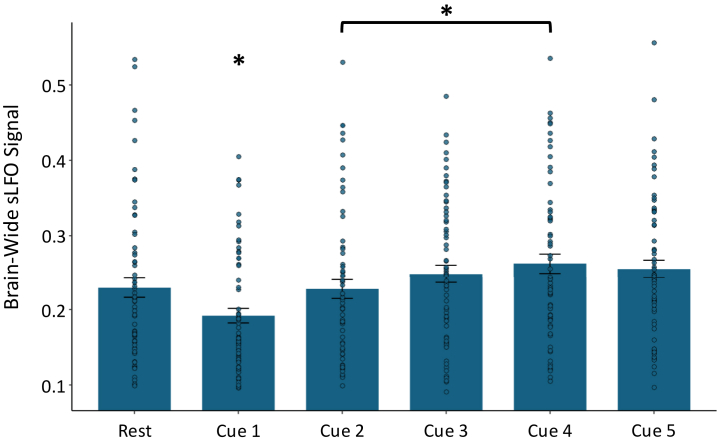
The systemic low-frequency oscillation (sLFO) at rest and across the cue reactivity task in the Cigarette Cue Reactivity Cohort. The plot depicts the significant main effect of scan block on sLFO (*F*_5,63_ = 11.32, *p* < .001). The brainwide average sLFO during cue block 1 was significantly lower than at rest (*t*_67_ = 3.18, *p* = .002) and lower than during all subsequent cue blocks (*t*_67_ = 4.08_cue 2_, 5.38_cue 3_, 6.59_cue 4_, 5.70_cue 5,_*p* < .001). Cue block 2 also showed significantly lower sLFO compared with cue block 4 (*t*_67_ = 3.27, *p* = .002). No other pairwise comparisons between scan blocks were significant. ∗*p* < .05.

There was a significant main effect of scan block on brainwide average sLFO in the Cigarette Cue Reactivity Cohort (*F*_5,63_ = 11.32, *p* < .001) (Figure 2). Post hoc paired *t* tests revealed that the brainwide average sLFO at cue block 1 was significantly lower relative to rest (*t*_63_ = −3.13, *p* = .002) and relative to any of the other cue blocks (*t*_63_ = 4.08_cue 2_, 5.38_cue 3_, 6.59_cue 4_, 5.70_cue 5,_ *p* < .001). Cue block 2 was also significantly lower than cue block 4 (*t*_63_ = 3.20, *p* = .002). No other differences were noted.

### The sLFO Is Related to Nicotine Dependence and Craving

**Figure 3:**
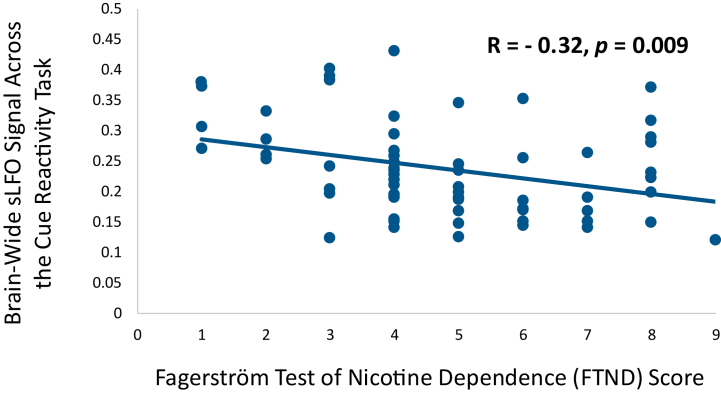
The relationship between nicotine dependence and the systemic low-frequency oscillation (sLFO) during the cue reactivity task completed by the Cigarette Cue Reactivity Cohort. FTND scores were significantly negatively correlated with the brainwide average sLFO across the 5 blocks of the cue reactivity task (*r* = −0.32, *p* = .009), indicating that more severe nicotine dependence was associated with lower sLFO during the task. However, FTND scores were not significantly correlated with the sLFO at rest (*r* = −0.047, *p* = .71).

**Figure 4:**
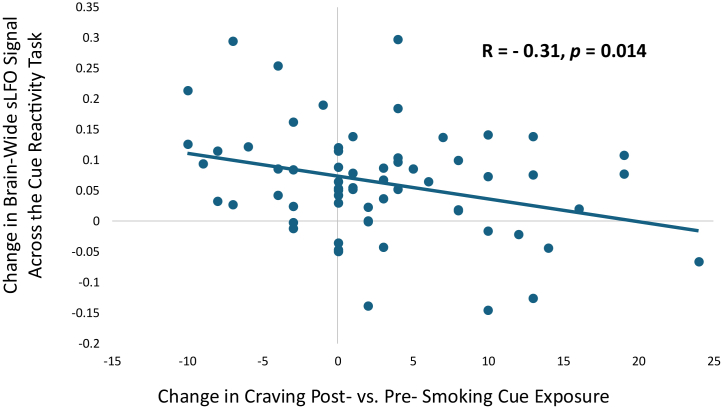
The relationship between cue-induced changes in subjective craving and the systemic low-frequency oscillation (sLFO) during the cue reactivity task completed by the Cigarette Cue Reactivity Cohort. The magnitude of nicotine craving increase during the cue reactivity task (post- > presmoking cue exposure) was significantly negatively correlated with the change in brainwide average sLFO strength (cue block 5 sLFO − cue block 1 sLFO) (*r* = −0.31, *p* = .014).

In the Cigarette Cue Reactivity Cohort, FTND scores were negatively correlated with the brainwide average sLFO when averaged across the cue reactivity task (*r* = −0.324, *p* = .009) (Figure 3). At rest, the sLFO and FTND scores were unrelated (*r* = −0.047, *p* = .71), replicating a similar null finding in the Chronic Nicotine Use and Matched Controls Cohort (*r* = 0.04, *p* = .77). The magnitude by which nicotine craving increased during the cue reactivity task was significantly correlated with the magnitude by which the brainwide average sLFO decreased during the task (*r* = −0.31, *p* = .014) (Figure 4). The relationship between cue-induced craving and the cue-induced change in sLFO remained when controlling for the sLFO during the first cue block (*r* = −0.26, *p* = .04).

### sLFO and Brain Reactivity to Smoking > Neutral Cues

**Figure 5:**
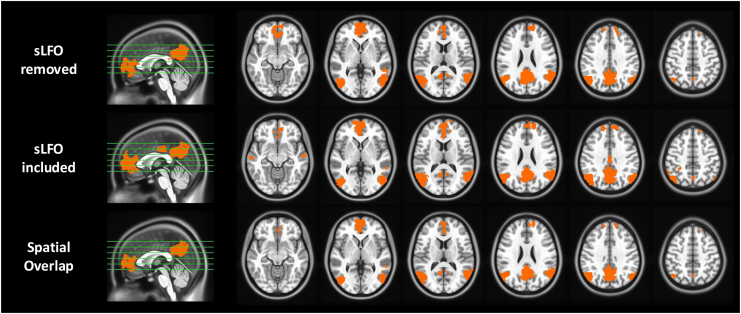
Smoking vs. neutral cue reactivity in the cigarette Cue Reactivity Cohort with the systemic low-frequency oscillation (sLFO) removed (top row), with the sLFO included (middle row), and their spatial overlap (bottom row). For both processing streams, *z* > 3.1, *p*_*corrected*_ = .05, and activation maps showed spatial overlap with *r* = 0.81.

During the cue reactivity task in the Cigarette Cue Reactivity Cohort, reactivity to smoking > neutral cues was significantly greater in brain regions including the medial prefrontal cortex, posterior cingulate cortex (PCC), precuneus, and bilateral angular gyrus (*z* > 3.1, *p*_*corrected*_ = .05) (Figure 5). This activation pattern was the same for data with and without the sLFO, as the results demonstrate spatial overlap with *r* = 0.81. There was no relationship between the brainwide average sLFO across the cue reactivity task and the smoking > neutral activation within the DMN (*r* = 0.14, *p* = .259) or insula (*r* = −0.10, *p* = .417). There was no change in the DMN (*F*_4,63_ = 1.55, *p* = .187) or insula (*F*_4,63_ = 0.61, *p* = .66) reactivity to smoking > neutral cues over time.

### sLFO Increases During Abstinence Relative to Satiety

**Figure 6:**
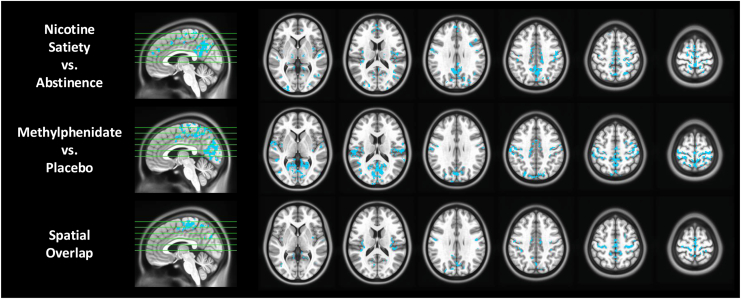
The voxelwise systemic low-frequency oscillation (sLFO) at rest during nicotine satiety vs. abstinence in the Chronic Nicotine Use and Matched Controls Cohort (top row), acute methylphenidate (MPH) administration vs. placebo in the Acute Psychostimulant Administration Cohort (middle row), and their spatial overlap (bottom row). The voxelwise sLFO decreased significantly during nicotine satiety relative to abstinence and during acute MPH administration relative to placebo. These reductions in the sLFO showed spatial overlap in regions between the superior sagittal sinus, inferior sagittal sinus, medial primary motor cortex, and precuneus (*p* < .001, cluster size > 160 mm^3^).

No significant difference was found between the sated nicotine-using group and the healthy control group in brainwide average sLFO in the Chronic Nicotine Use and Matched Controls Cohort (*t*_129_ = −0.705, *p* = .482). A voxelwise comparison between the groups also revealed no differences. In contrast, the abstinent nicotine-using group exhibited a significantly greater brainwide average sLFO compared with the healthy control group (*t*_99_ = −3.26, *p* = .002). Consistent with this finding, voxelwise analyses revealed that the sLFO increased significantly in abstinent individuals who smoked relative to healthy control individuals in the PCC (*p*_*corrected*_ < .001, cluster size > 160 mm^3^) (Figure S2). In the nicotine-using group, the brainwide average sLFO decreased significantly during nicotine satiety compared with abstinence (*n* = 65; *t*_64_ = −4.51, *p* < .001) (Figure S3). Relative to abstinence, the voxelwise sLFO decreased significantly during satiety in regions between the superior and inferior sagittal sinus, such as the medial primary motor cortex (mM1) and precuneus (*p*_*corrected*_ < .001, cluster size > 160 mm^3^) (Figure 6).

### Scan Order Does Not Impact the sLFO

No significant difference in brainwide average sLFO was found between visits in the HCP Cohort (*t*_457_ = 0.016, *p* = .9908). The voxelwise sLFO confirmed this null finding.

### Effects of Acute Psychostimulant Administration on the sLFO

Relative to placebo, acute nicotine had no impact on the sLFO at rest either at the brainwide average (*t*_58_ = −0.521, *p* = .604) or the voxelwise level in the Acute Psychostimulant Administration Cohort. Compared with placebo, methylphenidate significantly decreased the brainwide average sLFO at rest (*t*_58_ = −3.434, *p* = .001) (Figure S4). This decrease was due to a reduction in the voxelwise sLFO in brain regions between the superior and inferior sagittal sinus, such as the mM1, precuneus, and cuneus (*p*_*corrected*_ < .001, cluster size > 160 mm^3^) (Figure 6).

**Figure 7:**
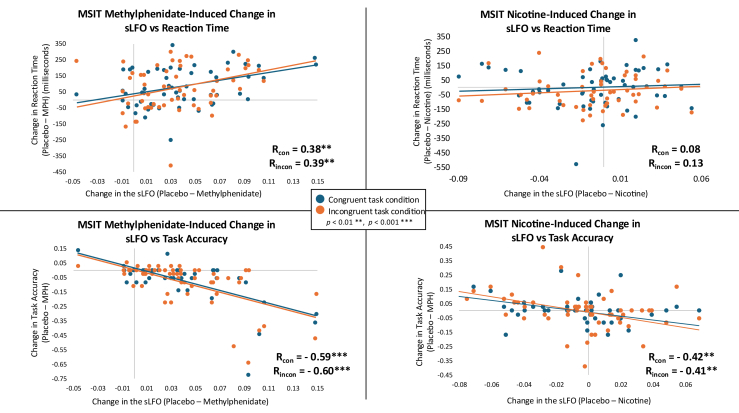
Correlations between the Multi-Source Interference Task (MSIT) drug-induced change in the brainwide average systemic low-frequency oscillation (sLFO) vs. reaction time and task accuracy in the Acute Psychostimulant Administration Cohort. The methylphenidate (MPH)-induced change in sLFO (placebo sLFO − MPH sLFO) was positively associated with the difference in reaction time (placebo − MPH) for both the congruent (con) (*r* = 0.38, *p* = .003) and incongruent (incon) (*r* = 0.39, *p* = .002) conditions and negatively associated with the difference in accuracy (placebo − MPH) for the congruent (*r* = −0.60, *p* < .001) and incongruent (*r* = −0.60, *p* < .001) conditions. There was no relationship between nicotine-induced changes in sLFO and reaction time for either the congruent (*r* = 0.07, *p* = .578) or incongruent (*r* = 0.08, *p* = .527) conditions. There was an association between nicotine-induced changes in sLFO and accuracy (placebo − nicotine) for the congruent (*r* = −0.35, *p* = .006) and incongruent (*r* = −0.39, *p* = .002) conditions.

A methylphenidate-induced change in the brainwide average sLFO during the MSIT (placebo − methylphenidate) was positively associated with the difference in reaction time (placebo − methylphenidate) for both the congruent (*r* = 0.38, *p* = .003) and incongruent (*r* = 0.39, *p* = .002) conditions and negatively associated with the difference in accuracy (placebo − methylphenidate) for the congruent (*r* = −0.60, *p* < .001) and incongruent (*r* = −0.60, *p* < .001) conditions (Figure 7). While there was no link between nicotine-induced changes in the voxelwise sLFO during the MSIT and reaction time for either the congruent (*r* = 0.07, *p* = .578) or incongruent (*r* = 0.08, *p* = .527) conditions, there was an association with accuracy (placebo − nicotine) for the congruent (*r* = −0.35, *p* = .006) and incongruent (*r* = −0.39, *p* = .002) conditions (Figure 7).

## Discussion

This study demonstrates that the sLFO carries clinically relevant information. First, we showed an impact of smoking cue exposure on the sLFO. Smoking cue exposure significantly reduced the brainwide average sLFO during the first cue reactivity block relative to rest. Prior work demonstrated that reductions in the brainwide average sLFO amplitude represent heightened physiological arousal (4), which is consistent with other work (5). Therefore, the sLFO reduction following cigarette-smoking cue exposure fits with the known arousal-inducing effect of drug-related cues (34, 35, 36, 37, 38). This is relevant as brain reactivity to smoking cues is linked to substance use, including treatment outcome (39,40), and subjective cue-induced craving has consistently been linked to drug use (41). In addition to cue-induced reductions, lower brainwide average sLFO during cue exposure was associated with greater nicotine dependence severity, suggesting that heightened physiological arousal during cue exposure relates to more severe dependence. Notably, no such relationship was observed at rest, indicating that associations between nicotine dependence and the sLFO are context dependent.

Following the initial cue-induced decrease in brainwide average sLFO, the signal increased across the smoking cue reactivity task, indicating a habituation of arousal-related physiology. Critically, smaller increases in the sLFO were associated with greater cue-induced craving, suggesting that sustained physiological arousal relates to stronger cue-induced craving. In contrast to these dynamic sLFO changes, brain reactivity to smoking > neutral cues remained constant across the task, indicating that brain reactivity to smoking cues persists despite sLFO habituation. Removing the sLFO from the fMRI signal did not alter whole-brain task activation or insula and DMN responses to smoking cues. This modest effect is expected, as task contrasts are robust to interindividual physiological variability. Collectively, these findings indicate a dissociation between sLFO reactivity and neural responses to smoking cues, suggesting that each provides complementary information.

Although different assessments of the sLFO during cue reactivity were related to craving and dependence, acute nicotine administration did not affect resting-state sLFO. Group differences between individuals who smoked and healthy control individuals were observed only during nicotine abstinence, not during satiety. Specifically, abstinent individuals exhibited higher sLFO relative to healthy control individuals, consistent with reduced physiological arousal during abstinence (42). This pattern was mirrored in individuals who smoked, as the sLFO was significantly lower during abstinence relative to satiety. Together with the absence of day-order effects in the HCP dataset, these findings support a pharmacologically driven, state-dependent effect of nicotine on the sLFO in the context of chronic tobacco use. These results are also consistent with evidence that acute and chronic nicotine exposure exert distinct effects on cerebrovascular function; acute nicotine exposure increases vascular tone (43), whereas chronic nicotine exposure impairs cholinergic angiogenesis (1,44,45), providing a plausible mechanism for differential effects on the sLFO.

To test generalizability beyond nicotine, methylphenidate was included as a comparator psychostimulant as both drugs increase arousal and dopaminergic signaling via distinct mechanisms (46, 47, 48). Similar to the effects of nicotine satiety relative to abstinence, acute methylphenidate produced voxelwise sLFO reductions in overlapping brain regions predominantly within sensory areas, consistent with regions previously associated with physiological influences on the global signal (49, 50, 51, 52).

During the MSIT, methylphenidate-induced reductions in the sLFO were associated with faster reaction times and increased accuracy, indicating that decreased sLFO was linked to improved task performance. While acute nicotine did not impact resting-state sLFO, nicotine-induced sLFO reductions during the MSIT were similarly associated with increased accuracy. Collectively, these findings link psychostimulant-induced sLFO reductions to improved cognitive performance, suggesting that studies evaluating the sLFO may help predict behavioral consequences of pharmacological interventions. To build on this, future studies could examine more dynamic links between sLFO fluctuations and variance in task performance.

### Conclusions

These results place the sLFO within a growing literature demonstrating that low-frequency BOLD fluctuations reflect interactions among autonomic physiology, vascular dynamics, cerebrovascular biomechanics, sleep-wake state, broad network activity, and diffuse neuromodulatory processes (4,53, 54, 55, 56, 57, 58, 59). The sLFO is a systemic, state-sensitive BOLD signal, distinguished by its time-lagged structure and strong autonomic-vascular contributions but influenced by multiple factors. For example, individuals who smoked did not differ from healthy control individuals during nicotine satiety but differed during nicotine abstinence. Because the same nicotine-using individuals showed changes in the sLFO across satiety and abstinence, these findings indicate that resting-state sLFO reflects state-dependent physiological changes. In contrast, prior work has demonstrated sLFO differences in populations with vascular or autonomic disruptions (60, 61, 62), indicating that the sLFO can capture both state- and trait-level effects. However, in this study, which centered on generally healthy individuals, the sLFO was sensitive to physiological state changes induced by pharmacology and task effects.

While more research is needed to generalize findings beyond our samples, our results are applicable across contexts. The results demonstrate that the sLFO is sensitive to pharmacological manipulation and emotionally evocative stimuli and is associated with clinical variables and cognitive performance. These effects are consistent across datasets and align with existing knowledge about the sLFO, supporting broader generalizability. Although we did not collect peripheral arousal measures (e.g., pupil diameter), recent work has demonstrated that the sLFO correlates with such indices with an *R* > 0.9 (4). Accordingly, this study builds on this foundation to evaluate the clinical utility of the sLFO. Moreover, given that the sLFO can be extracted from existing fMRI datasets without additional equipment, it provides a practical and scalable method for probing autonomic physiology. While the sLFO is not a direct neural signal, its vascular characteristics may reflect downstream physiological consequences of arousal-related neural processes. As a complement to neurovascular coupling measures, the sLFO contributes to the broader set of fMRI-based measures of arousal and brain health. Thus, isolating the sLFO both improves the specificity of neuronal signals in fMRI (4) and provides clinically relevant insights into pharmacological effects and individual differences, thereby expanding the clinical utility of neuroimaging.

## Supplementary Material

Supplemental Methods, Results, Figures S1–S4, Tables S1–S4

Key Resources Table
