# Supplementary material for: Functional Magnetic Resonance Imaging Signal Typically Viewed as “Noise” Has Clinical Relevance in Psychiatry
Source: Biol Psychiatry Glob Open Sci. 2026 Mar 6;6(3):100720. doi: 10.1016/j.bpsgos.2026.100720 (PMC13090997; doi:10.1016/j.bpsgos.2026.100720)
Supplement: Supplemental Methods, Results, Figures S1–S4, Tables S1–S4 [file mmc1.pdf]

SUPPLEMENTARY INFORMATION

Functional Magnetic Resonance Imaging Signal Typically Viewed as "Noise" Has Clinical Relevance in Psychiatry

Welsh *et al.*

Supplement

Participants

Cigarette Cue Reactivity Cohort. Clinical Trial Name & Number: Electronic Cigarettes and Reactivity to Smoking Cues, NCT01782599

The analyses presented here focus on data collected during the pre-treatment baseline assessment when all participants were still regularly smoking prior to the session. Participants were excluded if they met criteria defined by the Diagnostic and Statistical Manual of Mental Disorders (Fifth Edition) for moderate-severe drug or alcohol dependence, recent major depressive disorder (past 6 months), history of psychotic or bipolar disorder, serious medical illness, or pregnancy. All participants tested negative for current drug or alcohol use (excluding nicotine), and females tested negative for pregnancy. All procedures were completed at Harvard Medical School's McLean Hospital and were approved by the Partners Human Research Committee. Accrual began in October of 2015 and completed in April of 2021.

Table S1.

Demographic Characteristics of the Cigarette Cue Reactivity Cohort.

**Demographics**	
	**Individuals Who Use Nicotine (N = 64)**
Age (years)	29.19 (*SD* = 6.93)
Sex (male/female)	39/25
Race (*n*)	
Black/African American	6
White/Caucasian	47
Asian	7
More Than One Race	4
Ethnicity (*n*)	
Hispanic	6
Non-Hispanic	58
Education (years)	14.99 (*SD* = 2.01)

Demographic characterization of the Cigarette Cue Reactivity Cohort.

Chronic Nicotine Use and Matched Controls Cohort. Clinical Trial Name & Number: Imaging Biomarker for Addiction Treatment Outcomes, NCT03427424

All participants were recruited for a study investigating differences between individuals who did and did not use nicotine, with those in the nicotine-using group being offered enrollment in an individualized, counselor-involved treatment study following their initial study visit. No participants reported current psychiatric or neurological disorders as assessed by the Diagnostic and Statistical Manual of Mental Disorders (Fourth Edition). All participants tested negative for current drug or alcohol use (excluding nicotine for those in the nicotine-using group), and females tested negative for pregnancy. All procedures were completed at the National Institute on Drug Abuse and were approved by the institutional review board of the National Institutes of Health. Accrual began in October of 2013 and completed in June of 2023.

Table S2.

Demographic Characteristics of the Chronic Nicotine Use and Matched Controls Cohort.

**Demographics**	
	**Individuals Who Use Nicotine (N = 97)**
Age (years)	49.23 (*SD* = 10.84)
Sex (male/female)	55/42
Race (*n*)	
Black/African American	47
White/Caucasian	43
Asian	4
Native Hawaiian/Other Pacific Islander	1
More Than One Race	2
Ethnicity (*n*)	
Hispanic	4
Non-Hispanic	93
Unknown or not reported	0
Education (years)	13.41 (*SD* = 2.64)
	**Healthy Controls (N = 34)**
Age (years)	47.24 (*SD* = 9.45)
Sex (male/female)	22/12
Race (*n*)	

Black/African American	21
White/Caucasian	12
Asian	0
Native Hawaiian/Other Pacific Islander	0
More Than One Race	1
Ethnicity (*n*)	
Hispanic	1
Non-Hispanic	32
Unknown or not reported	1
Education (years)	13.56 (*SD* = 2.00)

Demographic characterization of the Chronic Nicotine Use and Matched Controls Cohort (N=131; N=97 individuals who use nicotine, N =34 healthy controls).

HCP Cohort.

All participants had no prior history of major psychiatric, neurological, or medical disorders known to affect brain function. Individuals were excluded from the analysis if they reported a family history of schizophrenia, met DSM-IV criteria for alcohol dependence, or had a lifetime history of significant substance use, defined as more than ten instances of cocaine, hallucinogen, opiate, sedative, or stimulant use; more than 20 instances of tobacco use; or more than 100 instances of marijuana use. Additionally, participants provided a breath sample indicating a blood alcohol content of less than 0.05 on the day of the scan, and a urine sample that tested negative for drugs of abuse (cocaine, marijuana, opiates, amphetamines, or methamphetamines). All procedures were approved by the Institutional Review Board at Washington University in St. Louis. Data collection took place at Washington University in St. Louis, Missouri. Accrual began in August of 2012 and completed in March of 2015.

Table S3.

Demographic Characteristics of the HCP Cohort.

**Demographics**	
	**Healthy Controls (N = 462)**
Age (years)	28.66 (*SD* = 3.65)
Sex (male/female)	181/281
Race (*n*)	
Black/African American	64

White/Caucasian	351
Asian/Native Hawaiian/Other Pacific Islander	35
More Than One Race	6
Unknown or Not Reported	6
Ethnicity (*n*)	
Hispanic	37
Non-Hispanic	418
Unknown or not reported	7
Education (years)	15.34 (*SD*= 1.59)

Demographic characterization of the HCP Cohort.

Acute Psychostimulant Administration Cohort. Clinical Trial Name & Number: Brain Networks and Addiction Susceptibility, NCT01924468

Exclusion criteria included, but were not limited to, a history of psychiatric disorders, HIV-positive status, or neurological illnesses. Medications that led to exclusion included, but were not limited to, benzodiazepines, barbiturates, anticonvulsants, antipsychotics, antidepressants, cold medications, and certain herbal supplements (e.g., Kava, Gingko biloba). All procedures were completed at the National Institute on Drug Abuse and were approved by the institutional review board of the National Institutes of Health. Accrual began in August of 2013 and the study completed in September of 2018.

Sample size was determined by power analysis with the key power analysis pertaining to genotypic and fMRI data – target accrual assured that analysis fall into range to detect expected signal changes when accounting for intra-participant variance. This equaled 80 participants total – with 86 actual enrollees to account for drop out. Notably, this sample size was based in part by the genotypic analysis which was not of interest to the present study. The present sample size (N=58) is within standard range for the present study question using fMRI data.

This work was part of a larger trial where the primary goal was evaluating how genotype and catecholaminergic function relates to brain network activity during task and rest. This was assessed by measuring genotype information and measuring how modulation of the dopaminergic and nicotinic system via pharmacological manipulation alters brain network activity at rest and during cognitive tasks. The field has since largely moved past the genotypic analysis which informed the original primary objective, while new fMRI techniques have emerged for understanding brain activity, including retrospective extraction of the sLFO signal. Here, we focused on the effect of pharmacology on the sLFO signal and assessed how such effects contribute to cognitive task performance.

Table S4.

Demographic Characteristics of Acute Psychostimulant Administration Cohort.

Demographics	
	**Healthy Controls (N = 58)**
Age (years)	31.17 (*SD* = 9.37)
Sex (male/female)	17/41
Race (*n*)	
Black/African American	10
White/Caucasian	37
Asian	5
More Than One Race	6
Ethnicity (*n*)	
Hispanic	9
Non-Hispanic	48
Unknown or not reported	1
Education (*n*)	
Less than High School	2
High School Complete/GED	8
Some / Partial Post-High School	21
College Graduate/Bachelor's Degree	18
Master's Degree	5
Professional Degree (MD, JD, Ph.D.)	4

Demographic characterization of the Acute Psychostimulant Administration Cohort.

fMRI data acquisition

Cigarette Cue Reactivity Cohort. All participants smoked one of their own cigarettes 1.5 hours prior to the start of MRI scanning to standardize the time since a cigarette was last smoked. Data were acquiring on a Siemens Prisma 3T Scanner (Erlangen, Germany) with a 64-channel head coil. The total scanning session included a 6-minute resting-state acquisition prior to the five, 5-minute runs of the cue reactivity task. Functional scans acquisition used the following parameters: TR = 0.72 s, TE = 30 ms, slices = 66, phase encode direction posterior to anterior, flip angle = 66°, voxel size = 2.5 mm isotropic, GRAPPA acceleration factor = 2, multi-band acceleration factor = 6. Following the functional scans, structural images were acquired with the following parameters: TR = 2.53 s, TE1 = 3.3 ms, TE2 = 6.98 ms, TE3 = 8.79 ms, TE4 = 10.65, flip angle = 7°, resolution= 1.33 x 1.0 x 1.0 mm.

Standard preprocessing was conducted using tools from the FMRIB software library (FSL) (www.fmrib.ox.ac.uk/fsl), including motion correction with MCFLIRT, brain extraction using BET, slice timing correction, spatial smoothing with a full-width half-maximum Gaussian kernel = 6 mm and high-pass filtering at 0.01 Hz.

Chronic Nicotine Use and Matched Controls Cohort. On the sated visit day, the participants in the nicotine-smoking group were instructed to smoke *ad lib* prior to MRI scanning. During the abstinence scan day, participants had abstained from smoking for at least 48 hours and as confirmed with a significant reduction in carbon monoxide levels (2.63 $\pm$ 1.90 ppm; $t(64) = 14.79$, $p < 0.001$). Data were acquired on a Siemens Trio 3T Scanner (Erlangen, Germany) using a 12-channel head coil. The total scanning session included an 8-minute resting state MRI scan. Functional scan acquisition used the following parameters: TR = 2.0 s, TE = 27 ms, flip angle = 78°, resolution = 3.44 mm x 3.44 mm x 4 mm. Following the resting state scan, structural images were acquired using the following parameters: TR = 1.9 s, TE= 3.51 ms, flip angle = 9°, resolution: 1.0 x 1.0 x 1.0 mm.

To preprocess the data, raw MRI DICOM data were converted to the standard BIDS structure with bidskit (https://github.com/jmtyszka/bidskit). Data were preprocessed using fMRIPrep (v.23.1.4, https://fmriprep.org/en/23.1.4/), which included discarding the first 2 volumes to account for the T1 non-equilibrium, slice timing correction, volume registration, head motion estimation, and spatial normalization to MNI space.

HCP Cohort. The opensource HCP minimal preprocessed resting-state fMRI data was used in this work. Data were acquired on a Siemens 3T Skyra scanner (Erlangen, Germany) modified to achieve a maximum gradient strength of 100 mT m^{-1} using a 32-channel head coil. Participants underwent two resting-state scans occurring on different days. Data were acquired across four, 14.4-minute resting state runs—two runs were completed during one visit and two in a separate visit. Within each session, oblique axial acquisitions alternated between phase encoding in a right-to-left (RL) direction during one run and phase encoding in a left-to-right (LR) direction during the other run. Functional scan acquisition used the following parameters: TR = 0.72 s, TE = 33.1 ms, flip angle = 52°, field of view = 280 x 180 mm^2, matrix = 140 x 90, echo spacing = 0.58 ms, bandwidth = 2,290 Hz px^{-1}. Slice thickness was set to 2.0 mm, 72 slices and 2.0 mm isotropic voxels, multiband acceleration factor = 8.

Acute Psychostimulant Administration Cohort. All participants underwent scanning across three drug conditions, which were administered on separate days in a double-blind placebo-controlled design: placebo/placebo, placebo/nicotine, and placebo/methylphenidate. The drug doses were 7 mg of transdermal nicotine (patch) and 20 mg oral methylphenidate. Each scanning session was identical and took place at the peak drug effect: 2 hours post-nicotine and 1 hour post-methylphenidate/placebo, timed according to absorption rates to ensure high and stable plasma levels of medication during the scan. To maintain blinding, participants received either drug or placebo twice on a given scan day—once at the nicotine timepoint and once at the

methylphenidate time point. On the placebo scan day, both timepoints involved the administration of placebo. Participants only received one active drug on each study day and placebo at the other timepoint. If participants were discontinued, slots were reused to maintain the balanced randomization. The study physician generated this randomization and assigned participants to interventions. The study physician and lead associate investigators were responsible for enrolling participants. All 58 subjects included in our analysis completed all sessions and did not having missing data.

Data were acquired on a Siemens Trio 3T scanner (Erlangen, Germany) using a 12 channel head coil. The total scanning session included an 8-minute resting-state acquisition prior to the three, approximately 11-minute blocks of the Multi-Source Interference Task (MSIT). Functional scan acquisition used the following parameters: TR = 2 s, TE = 27 ms, flip angle = 78°, resolution = 3.4375 mm x 3.4375 mm x 4 mm. Following the resting state scan, structural images using were acquired using the following parameters: TR = 1.9 s, TE= 3.51 ms, flip angle = 9°, resolution: 1.0 x 1.0 x 1.0 mm.

To preprocess the data, raw MRI DICOM data were converted to the standard BIDS structure with bidskit (https://github.com/jmtyszka/bidskit). Data were preprocessed using fMRIPrep (v.22.1.0, https://fmriprep.org/en/22.1.0/), which included discarding the first 2 volumes to account for the T1 non-equilibrium, slice timing correction, volume registration, head motion estimation, and spatial normalization to MNI space.

Systemic Low Frequency Oscillation (sLFO) Identification

The sLFO signal is extracted and iteratively refined from the BOLD fMRI data, as described in detail previously (Tong *et al.*, 2019). Briefly, an initial candidate sLFO signal (typically the global mean BOLD signal) is cross-correlated with each voxel time course to estimate voxel-wise blood arrival times. The low-frequency component of each voxel's signal is then temporally aligned by applying a negative timeshift equal to its estimated delay. A refined sLFO reference signal is computed from the aligned voxel signals using either principal component analysis or a weighted average. This procedure is repeated until convergence, which typically occurs within three iterations.

Cigarette Cue Reactivity Analyses

Following preprocessing of the fMRI data from the cigarette cue reactivity task, the first-level analysis was conducted on each of the participant's five task runs. The first-level general linear model included regressors convolved with the gamma hemodynamic response function corresponding to smoking, neutral, and target images. Confound regressors modeled motion (*x*, *y*, *z* translation and rotation) and spikefix-identified artifacts. The smoking > neutral image

contrast was conducted. First-level results were then combined (across task blocks) using a second-level fixed effects analysis to generate the average brain reactivity for each individual participant. The group-level analysis was then conducted using FLAME (FMRIB's Local Analysis of Mixed Effects) to identify average brain reactivity across all participants corrected for multiple comparisons using a cluster threshold of $Z = 3.1$, $p < 0.05$.

Supplemental Figures

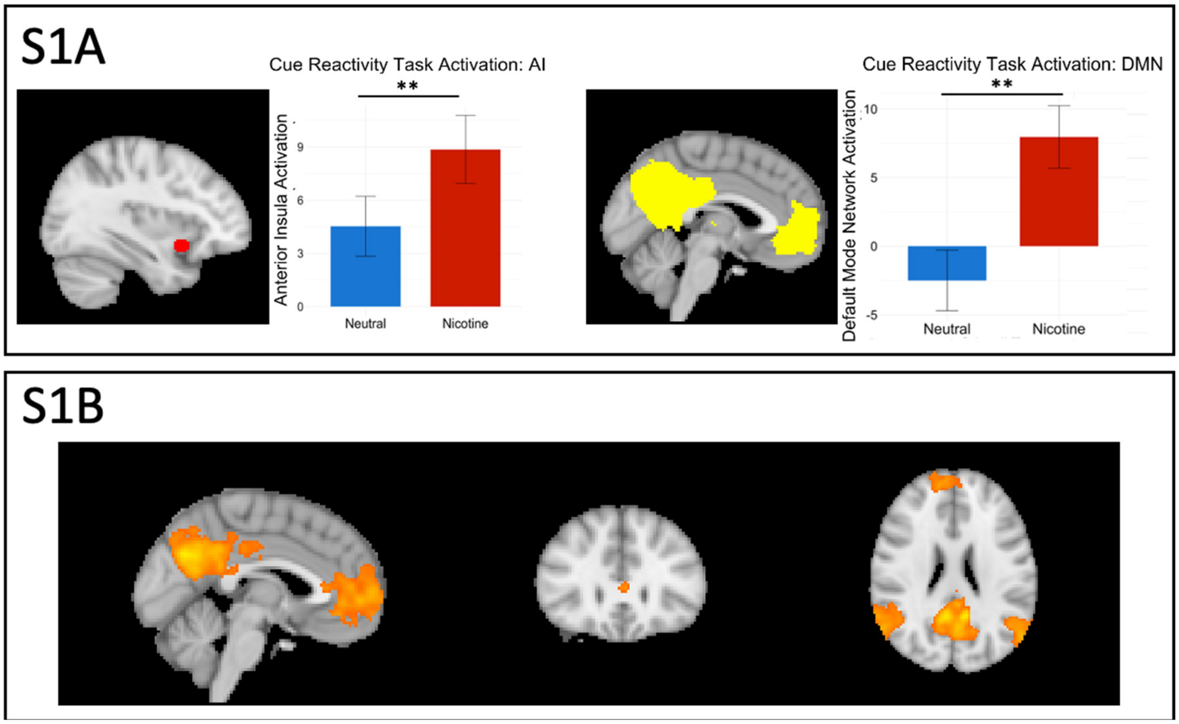

Supplemental Figure 1: Cue reactivity in the insula and Default Mode Network using BOLD data with the sLFO removed in the Cigarette Cue Reactivity Cohort. S1A: Depict the regions of interest (Adapted from Murray *et al.*, 2025) defined for the anterior insula and default mode network, which overlap with known whole brain activation patterns (S1B). There was no relationship between the brain-wide sLFO across the cue reactivity task and the smoking > neutral activation within the DMN (r = -0.18; 95% CI [-0.41, 0.07]; $p = 0.259$) or insula (r = -0.10; 95% CI [-0.34, 0.15]; $p = 0.417$). *** $p < 0.01$

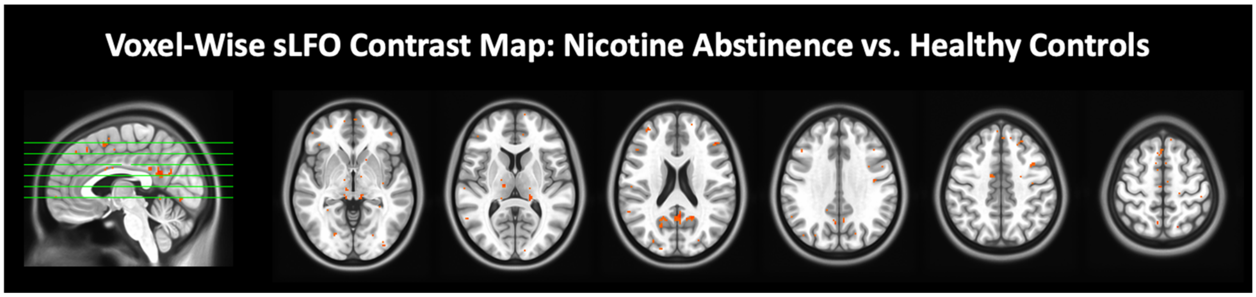

Supplemental Figure 2: Voxels showing significant increases in the voxel-wise sLFO during nicotine abstinence in individuals who smoke relative to healthy controls in the Chronic Nicotine Use & Matched Controls Cohort. The sLFO signal was significantly greater for those experiencing nicotine abstinence relative to healthy controls in the posterior cingulate cortex ($p < 0.001$, cluster size > 160 mm^3).

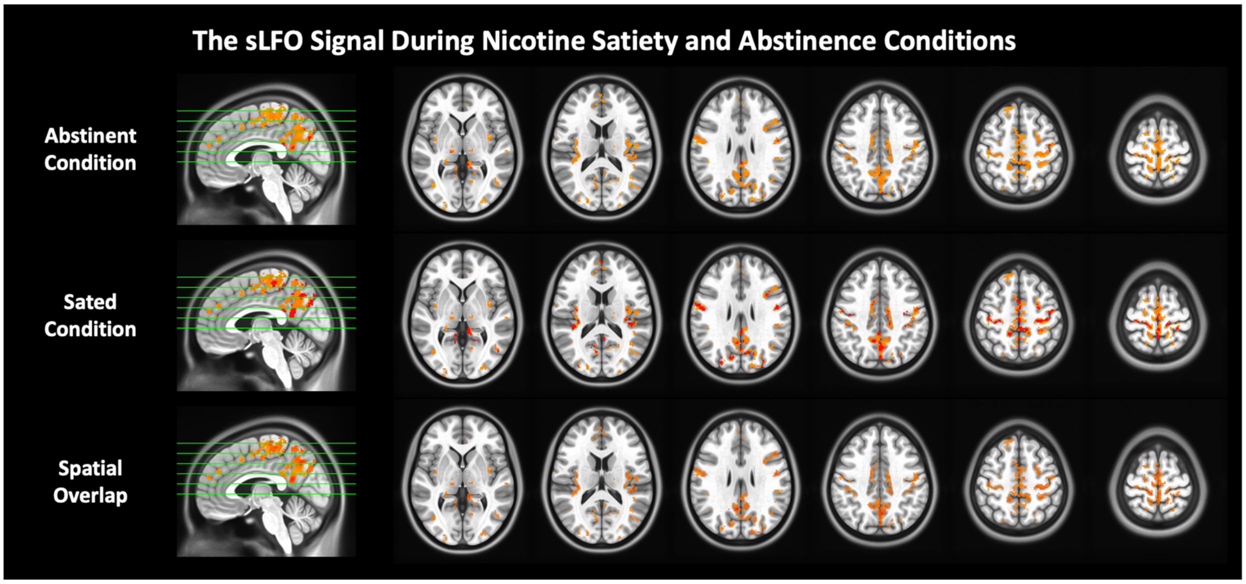

Supplemental Figure 3: Brain regions showing significant increases in sLFO signal during nicotine abstinence relative to satiety in those who use nicotine chronically in the Chronic Nicotine Use & Matched Controls Cohort. The sLFO signal significantly increased during abstinence (top row) relative to satiety (middle row) in regions between the superior sagittal sinus, inferior sagittal sinus, medial primary motor cortex (mM1), and precuneus ($p < 0.001$, cluster size > 160 mm³; bottom row).

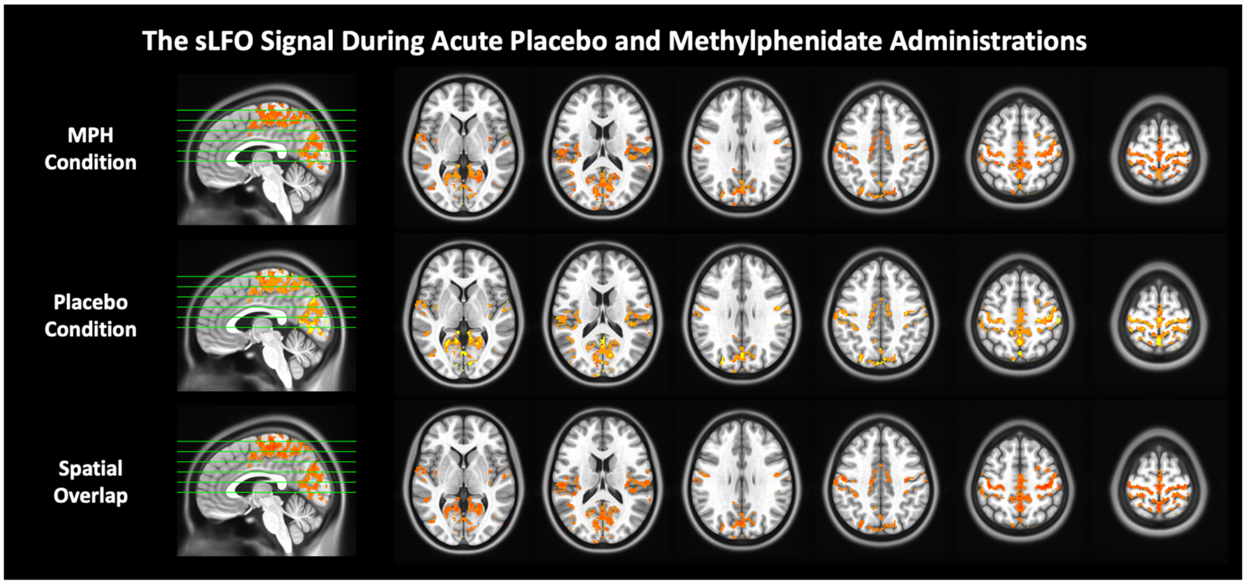

Supplemental Figure 4: Brain regions showing significant decreases in sLFO signal during acute methylphenidate administration relative to placebo at rest in the Acute Psychostimulant Administration Cohort. The sLFO signal significantly decreased under methylphenidate (MPH; top row) compared to placebo (middle row) in regions between the

superior sagittal sinus, inferior sagittal sinus, medial primary motor cortex (mM1), precuneus, and cuneus ($p < 0.001$, cluster size > 160 mm^3; bottom row).

References

1. Tong Y, Hocke LM, Frederick BB (2019): Low frequency systemic hemodynamic "noise" in resting state BOLD fMRI: characteristics, causes, implications, mitigation strategies, and applications. *Frontiers in neuroscience*. 13:787.
2. Murray, L., *et al.* Brain reactivity to nicotine cues mediates the link between resting-state connectivity and cue-induced craving in individuals who smoke or vape nicotine. *Neuropsychopharmacology*, 1-8 (2025).

HCP Accession Numbers

100206	127731	154734	182739	237334	412528	620434	833148
100307	127832	154835	185139	245333	415837	622236	833249
100610	128127	155635	185846	246133	419239	626648	835657
101309	128632	155938	185947	248339	422632	628248	841349
102311	128935	156435	186040	250427	432332	634748	849264
102715	129028	156536	187143	250932	436845	638049	852455
102816	129129	156637	188448	263436	441939	645551	856766
103414	129331	157336	188549	281135	448347	647858	856968
103818	130013	157437	189349	283543	453441	654350	859671
104012	130518	158035	191235	285446	456346	656657	861456
105014	130619	159441	191942	286347	459453	657659	865363
105923	130821	161630	192035	286650	463040	660951	871762
106016	130922	162026	192540	287248	469961	662551	872562
106521	131217	162228	192843	289555	475855	668361	872764
106824	132017	162733	193239	293748	495255	671855	873968
107422	132118	163129	193845	297655	499566	673455	877168
108222	133019	163432	194140	298051	510326	675661	877269
108323	133625	164030	194443	304020	512835	680250	878776
108525	134223	164131	195041	304727	513130	680452	878877
109325	135225	164939	196144	305830	513736	683256	880157
109830	135528	165638	196346	307127	516742	686969	887373
110411	135932	166640	197348	308129	518746	693764	888678
111009	136833	167238	198047	309636	522434	702133	889579
111413	137128	168139	198350	316633	529549	709551	891667
111716	137431	168341	198653	318637	529953	713239	894067
112314	137532	169444	199352	320826	530635	715647	894673
112920	137633	169545	200311	321323	531940	715950	894774
113215	138231	170631	200614	322224	540436	724446	899885
113619	138534	171532	201414	325129	547046	728454	901139
114217	139435	172029	201515	329844	552544	731140	904044
114621	139637	172332	201818	330324	553344	732243	912447
114823	141422	172433	202113	334635	555348	734045	917255
115017	142828	172534	203418	346137	555651	744553	917558
115320	143224	172938	204420	349244	558657	748662	919966
115825	144125	173334	204622	350330	562446	749058	922854
117021	144731	173435	205119	356948	566454	749361	926862

117122	144832	173536	205725	360030	567052	753251	930449
117324	145127	173738	205826	365343	567961	756055	932554
118528	145834	173839	206323	366446	568963	757764	933253
118831	146129	173940	206525	368551	570243	759869	937160
119126	146937	174437	206828	368753	571144	761957	942658
120111	147737	174841	208630	376247	572045	765864	943862
120515	148133	175136	209329	378756	573249	770352	951457
121416	149337	175439	209935	378857	573451	773257	952863
122317	149741	175540	210415	381543	576255	783462	955465
122822	150625	176441	211114	382242	580650	784565	958976
123117	151223	176542	211619	385046	581450	788674	965367
123420	151425	176845	211821	390645	585862	788876	966975
123521	151728	177746	212015	391748	586460	789373	969476
123723	151829	178142	212318	392750	588565	792564	970764
123925	152427	178243	213421	393247	594156	793465	979984
124422	153025	178849	213522	393550	598568	803240	984472
124826	153126	178950	214221	394956	601127	815247	987074
125424	153227	179245	214423	395251	604537	816653	992673
126426	153732	179346	224022	395756	614439	818455	993675
126628	154330	180230	228434	397760	615441	826353	996782
127327	154431	180735	231928	397861	615744	826454	
127630	154532	182032	233326	406432	616645	828862	
127731	154734	182739	237334	412528	620434	833148	
